# Discrimination of Structural and Immunological Features of Polysaccharides from Persimmon Leaves at Different Maturity Stages

**DOI:** 10.3390/molecules24020356

**Published:** 2019-01-19

**Authors:** Young-Ran Song, Ah-Ram Han, Tae-Gyu Lim, Ji-Hyun Kang, Hee-Do Hong

**Affiliations:** Korea Food Research Institute, Wanju-gun, Jeollabuk-do 55365, Korea; song.young-ran@kfri.re.kr (Y.-R.S.); lambo3@kfri.re.kr (A.-R.H.); tglim83@kfri.re.kr (T.-G.L.); kang.ji-hyun@kfri.re.kr (J.-H.K.)

**Keywords:** persimmon leaf, polysaccharide, immunostimulatory activity, harvest maturity

## Abstract

In this study, we investigated changes in the structural and immunological features of polysaccharides (S1-PLE0, S2-PLE0, and S3-PLE0) extracted from persimmon leaves at three different growth stages. Physicochemical analyses revealed that their chemical compositions, molecular weight distributions, and linkage types differed. High-performance size-exclusion chromatograms showed that the molecular weights of the polysaccharides increased during successive growth stages. In addition, seasonal variation of persimmon leaves affected the sugar compositions and glycosidic linkages in the polysaccharides. S2-PLE0 was composed of comparatively more galactose, arabinose, rhamnose, xylose, and galacturonic acid, showing the presence of β-glucopyranoside linkages. Significant differences also occurred in their immunostimulatory effects on RAW264.7 macrophages, with respect to which their activities could be ordered as S2-PLE0 > S3-PLE0 > S1-PLE0. Evidently, S2-PLE0 showed the greatest immunostimulatory activity by enhancing the phagocytic capacity and promoting nitric oxide (NO) and cytokines secretion through the upregulation of their gene expression in macrophages. These results suggest that differences in the structural features of polysaccharides according to the different maturity of persimmon leaves might impact their immunostimulatory properties. The results also provide a basis for optimizing persimmon leaf cultivation strategies for food and medical uses of the polysaccharides.

## 1. Introduction

Polysaccharides are important biological macromolecules that are widely distributed in natural products, including plants and microorganisms [1]. Many natural polysaccharides have attracted attention as highly valuable biomaterials owing to their biocompatibility, low toxicity, and biodegradable properties [2]. In particular, it has been shown that plant-derived polysaccharides have a wide range of pharmacological properties including antioxidant, antitumor, and immunomodulatory activities [1,3,4,5]. Of these functions, immunostimulation, is one of the most important actions of polysaccharides, characterized by their broad spectrum of immune activities, and mounting evidence has highlighted these macromolecules as ideal immunostimulants [1,4,6,7]. Immunostimulatory polysaccharides can stimulate host immune functions via direct or indirect interactions that trigger diverse cellular and molecular events, leading to immune system activation [6]. Various types of immune cells, such as monocytes, macrophages, dendritic cells, neutrophils, lymphocytes, and natural killer cells, can be activated by immunostimulatory polysaccharides [8]. Therefore, natural polysaccharides from plants are receiving increasing attention in the fields of therapeutics and functional foods.

The persimmon plant (*Diospyros kaki* Thumb.) is widely distributed in East Asian countries, such as China, Japan, and Korea. Recently, the global production of persimmon exceeded 5.0 million tons, accounting for 0.75% of global fruit production [9]. Persimmon fruit is commonly eaten fresh, dried, or cooked [10]. Persimmon leaves have been used in folk medicine and consumed in health-promoting beverages, particularly as a commercial tea in Asia [11]. Recently, the leaves have become increasingly popular as a natural food additive in the food, pharmaceutical, and cosmetic industries due to their functional properties, including their anti-oxidant, anti-diabetic, anti-tumor, and immunological effects [11,12,13,14]. These potential health benefits are attributed to bioactive compounds in the persimmon leaves. Many studies have been focused on low-molecular-weight phytochemicals in persimmon leaves, such as tannins, flavonoids, triterpenoids, and vitamin C [10,12,14,15,16]. However, in recent years, researchers have begun to investigate polysaccharides with relatively higher molecular weights in persimmon leaves. Persimmon leaf-derived polysaccharides have been shown to exert hypoglycemic, anti-tumor, anti-metastatic, and immunoregulatory effects [17,18,19,20]. Thus, polysaccharides are one of the main constituents of persimmon leaves that contribute to this plant’s bioactivities. Previously, we obtained an immunostimulatory polysaccharide fraction (PLE0) from persimmon leaves and demonstrated that the PLE0 fraction had immunostimulatory effects in a cyclophosphamide-induced, immunosuppressed animal model and in RAW264.7 macrophages by activating TLR2-mediated NF-κB and MAPKs signaling pathways [21,22]. The chemical properties of PLE0 from persimmon leaves were also characterized, indicating that the polysaccharides are a group of hetero-polysaccharides with different molecular weights of 11–59 kDa and consist mainly of galacturonic acid, arabinose, galactose, and rhamnose [22].

Many researchers have focused on extraction methods, as well as on the structural and pharmacological properties of plant-derived polysaccharides [3,4,5,7]. In particular, research on optimizing polysaccharide extraction from plant sources has garnered increased attention, since extraction techniques can significantly affect the yield, physicochemical properties, and biological activities of polysaccharides [13,23]. However, the impacts of the quality of the raw materials on the structural and biological characteristics of polysaccharides tend to be neglected. Indeed, the accumulation of phytochemicals in plants is affected by various factors, such as the cultivar, cultivation conditions, and harvesting time [24]. The harvesting time of plants has been considered especially important regarding the compositions and contents of their bioactive compounds [25,26]. Some studies have reported the seasonal variation of phytochemicals in persimmon leaves. The seasonal compositional change of flavonol glycosides in persimmon leaves collected at different growing times from April to October were elucidated, indicating that the flavonol glycosides were diversified, increased until June, and then were stable during later growth stages [16]. It was also demonstrated that the persimmon leaves harvested in June had the highest polyphenol content and α-amylase inhibitory activity among the leaves harvested at 11 different growing stages, [12]. In addition, persimmon leaves harvested in May had the highest amounts of phenolic compounds and flavonoids and the highest antioxidant activity among different harvesting times [27]. However, the seasonal variations of the characteristics of polysaccharides in plants (including their physicochemical and biological properties and yields) remain largely unexplored.

In this study, we aimed to elucidate seasonal changes in polysaccharides derived from persimmon leaves by analyzing their chemical and structural characteristics and immunostimulatory activities at different maturity stages. To our knowledge, this study represents the first attempt at evaluating seasonal variations in active polysaccharides during leaf development in plants.

## 2. Results and Discussion

### 2.1. Comparison of the Physicochemical Properties of Three PLE0s

#### 2.1.1. Chemical and Monosaccharide Compositions

In this study, three polysaccharide fractions (S1-PLE0, S2-PLE0, and S3-PLE0) were obtained from persimmon leaves cultivated to three different maturity stages. As shown in Table 1, the polysaccharide yields in persimmon leaves significantly differed according to the cultivation time, as follows: S1-PLE0 (1.80 ± 0.23%) > S2-PLE0 (1.44 ± 0.18%) > S3-PLE0 (1.20 ± 0.24%). The results suggested that the yields decreased with increasing cultivation times. The total carbohydrate contents of all three polysaccharides were 98.2% (S1-PLE0), 97.9% (S2-PLE0), and 97.7% (S3-PLE0), on the basis of analysis of their neutral sugar and uronic acid levels. Among the three polysaccharide fractions, S2-PLE0 contained the lowest neutral sugar (57.0%) and highest uronic acid (40.9%) levels. All three PLE0s showed minor levels of protein content. In addition, all three extracts showed similar levels of 2-keto-3-deoxy-mannooctanoic acid (KDO)-like materials. KDO is a unique component of the highly branched rhamnogalacturonan-II (RG-II) moiety of pectic polysaccharides found in some plants [6]. Thus, the three PLE0s may contain the RG-II moiety, but their levels did not appear significantly different (*p* > 0.05). Pectin comprises a group of carbohydrate polymers that help form primary cell walls, and the heterogeneous polysaccharides are considered the most structurally complex among the polysaccharides commonly found in plants [28]. As a group of acidic heteropolysaccharides, pectic polysaccharides are mainly composed of an α-(1-4)-linked linear homo-galacturonic (HG) backbone, alternating with regions of two types of highly branched rhamnogalacturonans (RG-I and RG-II) and/or xylogalacturonan (XGA), thereby containing a high proportion of galacturonic acid (~70%) [6]. RG-I is composed of a rhamnogalacturonan backbone with several side chains consisting of arabinose and galactose residues (e.g., arabinan, galactan, and arabinogalactans), whereas RG-II are branched structures composed of more diverse monosaccharides, including some rare sugars, such as apiose and KDO, which are usually not observed in other polysaccharides [2]. The biological activities of pectic polysaccharides are attributed to the RG-I and RG-II regions, rather than the HG region [19]. Pectic polysaccharides from persimmon leaves are mainly composed of rhamnose, arabinose, galactose, glucose, and xylose, with a high portion of galacturonic acid and they serve immunostimulatory, anti-tumor, and anti-metastatic roles by enhancing the activity of macrophages and natural killer (NK) cells [17,20,21,22]. Here, the sugar compositions of S1-PLE0, S2-PLE0, and S3-PLE0 were also determined by high-performance anion-exchange chromatography (HPAEC) (Table 1 and Figure S1), and seven neutral sugars and two uronic acids were detected in all samples. The major constituents in all PLE0s were galactose, arabinose, glucose, rhamnose, and galacturonic acid, indicating they have pectin-like structures, which is consistent with previous studies [17,21,22]. However, distinct molar ratios for galactose, arabinose, glucose, rhamnose, and galacturonic acid of 2.06:0.60:1.00:0.31:7.78 (S1-PLE0), 4.46:1.59:1.00:0.75:10.94 (S2-PLE0), and 4.07:0.94:1.00:0.11:3.95 (S3-PLE0), respectively, were observed in the PLE0 fractions. Galacturonic acid appeared to be the most abundant among all PLE0s, but the content was obviously higher in S2-PLE0, in agreement with the result of the uronic acid test. In addition, the galactose, arabinose, mannose, and glucuronic acid levels were significantly higher in S2-PLE0 and S3-PLE0 compared with S1-PLE0. S2-PLE0 contained the highest proportions of galactose, arabinose, rhamnose, xylose, and galacturonic acid, suggesting that S2-PLE0 might contain relatively more abundant or more complex RG-I side chains compared to S1-PLE0 and S3-PLE0 [28]. Some studies have indicated that the bioactive compounds of plants show significant variability in their chemical contents at different maturity stages [12,16,25,26,27]. Here, also, the chemical compositions of S1-PLE0, S2-PLE0, and S3-PLE0 from persimmon leaves at three different maturity stages varied significantly.

#### 2.1.2. Molecular Weight Distribution

**Figure 1:**
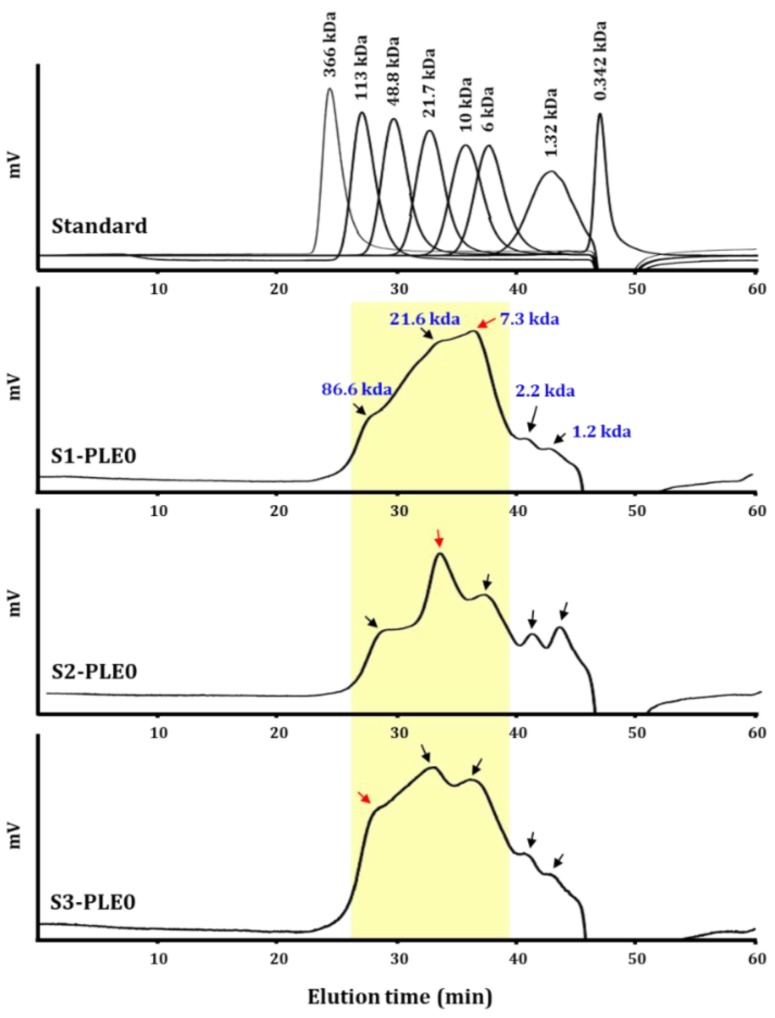
Comparison of the molecular weight patterns of S1-PLE0, S2-PLE0, and S3-PLE0 extracted from persimmon leaves at three different maturity stages.

The molecular weight of a polysaccharide is an important feature for understanding structure–function relationships [19]. Generally, plant-derived polysaccharides have molecular weights highly dispersed within a large range, from a few kilo-daltons to several mega-daltons [6]. Previously, the molecular weight values of three polysaccharides, which were extracted from persimmon leaves and purified by gel-permeation chromatography, were 11 kDa, 18 kDa, and 59 kDa [22]. In this study, the molecular weight distributions of polysaccharides extracted from persimmon leaves at three different stages were determined (Figure 1). The high-performance size-exclusion chromatography (HPSEC) chromatograms for S1-PLE0, S2-PLE0, and S3-PLE0 showed broad molecular weight distribution patterns, indicating they are polydisperse heteropolysaccharides. They were mainly composed of five distinct groups with average molecular weight values of 86.6 kDa, 21.6 kDa, 7.3 kDa, 2.2 kDa, and 1.2 kDa, as estimated on the basis of calibration with pullulan standards. However, the molecular weights of the most abundant portions in the polysaccharide fractions tended to increase with increasing growth periods of persimmon leaves: approximately 7.3 kDa in S1-PLE0, 21.6 kDa in S2-PLE0, and 86.6 kDa in S3-PLE0. In plants, specific differences in cell wall compositions can be observed during plant growth, enabling cell expansion, wherein polysaccharide backbone synthesis, backbone substitution with other sugars, and chain elongation and decomposition occurr [28]. Thus, polysaccharides deposited in the cell wall can vary structurally depending on the development stage, suggesting that during persimmon leaf development, polysaccharides might undergo synthesis and steady linking rather than decomposition.

#### 2.1.3. Linkage Analysis of Polysaccharides

**Figure 2:**
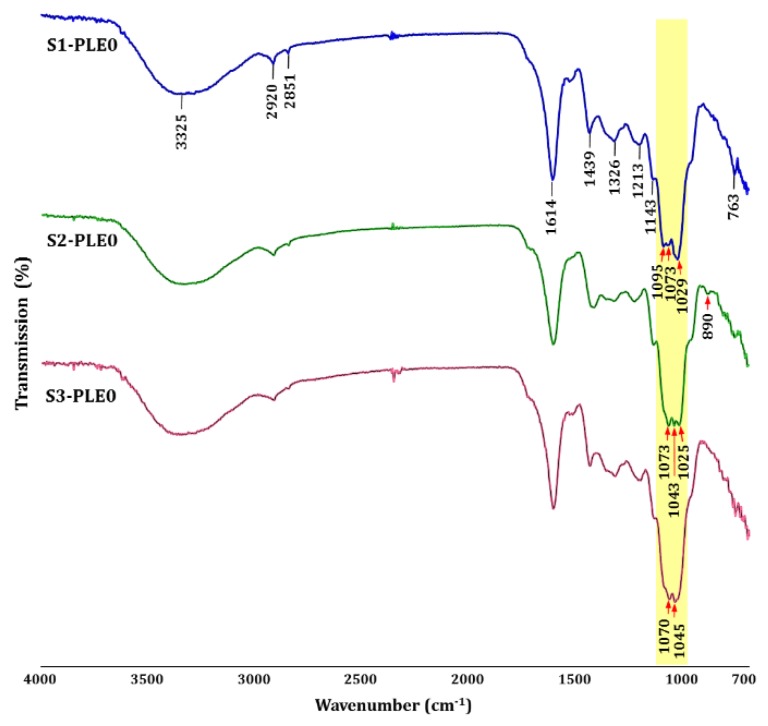
FT-IR spectra of S1-PLE0, S2-PLE0, and S3-PLE0 extracted from persimmon leaves at three different maturity stages.

Fourier-transform infrared spectroscopy (FT-IR) analysis is a powerful tool for identifying characteristic functional groups and linkage bonds in polysaccharides, clearly revealing characteristic absorption peaks of polysaccharides [29]. The FT-IR spectra for S1-PLE0, S2-PLE0, and S3-PLE0 are shown in Figure 2.

The three polysaccharides possessed similar absorption patterns, indicating typical glycosidic structures, except for the patterns of stretching peaks ranging from 1100 to 1000 cm^−1^. Specifically, the broad intense peak at approximately 3325 cm^−1^ was ascribed to the stretching vibration characteristic of hydroxyl groups (O–H), and the weak peaks at 2920 and 2851 cm^−1^ were characteristic of C–H bonds, such as in –CH_2_ and –CH_3_ [30]. The intense absorptions at 1614 and 1439 cm^−1^ were associated with asymmetric and symmetric stretching vibrations of COO^−^, respectively, revealing the presence of polygalacturonic acid in pectin polysaccharides [31], in agreement with the chemical composition results. The absorption peak at 1614 cm^−1^ could also be influenced by the bending vibration of bound water [3]. The absorption at 1326 cm^−1^ was derived from C–C and CH_2_ vibrations, and the peak at 1213 cm^−1^ was ascribed to the C–H-stretching vibration of pectic acid salt [31]. The high absorptions in the 1200–1000 cm^−1^ region represent specific bands typical of a pyranose ring vibration, which overlapped with the C–O–C glycosidic bond vibration (at 1143 cm^−1^) and the C–C, C–OH, and C–H vibrations, indicating the presence of pyranose monomers [4]. Specifically, polysaccharides strongly absorbed in the sugar region of 1100–1000 cm^−1^, indicating the presence of specific high bands assigned to pectins in the 1100–1000 cm^−1^ region; in fact, absorption patterns can be used to discriminate the sugar constituents of polysaccharides [29]. S1-PLE0, S2-PLE0, and S3-PLE0 displayed specific absorption characteristics of pectic polysaccharides, showing strong absorptions in the 1100–1000 cm^−1^ region; however, their stretching patterns were different. Our data also suggested that the three polysaccharides have different sugar compositions. Additionally, the characteristic peaks at 890 and 763 cm^−1^ were associated with the presence of β-pyranoside and α-d-glucopyranose linkages, respectively [3]. Here, only S2-PLE0 exhibited weak absorption at 890 cm^−1^, which probably resulted from linkages of various structural sugar elements in the branched area of the pectic polysaccharides, such as RG-I, RG-II, and XGA [28]. These findings are consistent with the results of the monosaccharide composition. Overall, these spectra confirmed that S1-PLE0, S2-PLE0, and S3-PLE0 possessed the typical absorption peaks of pectic polysaccharides, with similar backbones and chemical groups; however, their sugar compositions and pyranoside linkages differed according to the maturity stages of persimmon leaves. The harvest time of persimmon leaves significantly affected the physicochemical properties of polysaccharides; moreover, our findings suggest that the three polysaccharides exhibit different structural forms.

### 2.2. Comparing the Immunostimulatory Activities of the Three PLE0s

#### 2.2.1. Effects on Macrophage Viability and Phagocytic Ability

**Figure 3:**
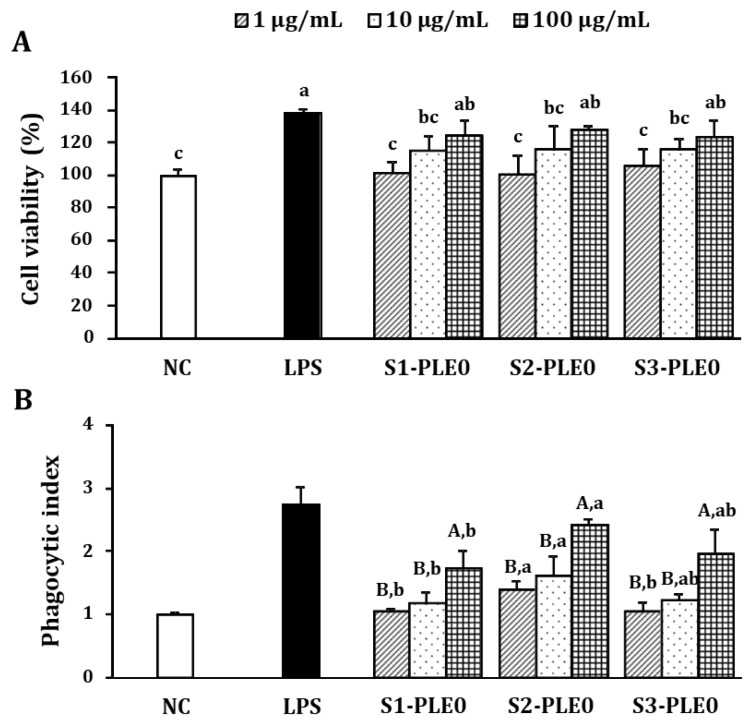
Cytotoxic effects (**A**) and phagocytic activities (**B**) of PLE0s extracted from persimmon leaves at three different maturity stages on RAW264.7 macrophages. RAW264.7 cells were treated with various concentrations of the polysaccharide fractions for 24 h. Cytotoxicity was determined by performing CCK-8 assays, and phagocytic activity was determined using a neutral red-uptake assay. Medium alone was used as the negative control (NC), and lipopolysaccharide (LPS) alone (1 µg/mL) was used as the positive control. Lowercase letters (a–c) expressed in (**A**) reflect significant differences (*p* < 0.05) among the tested samples. The uppercase letters (A,B) shown in (**B**) indicate significant differences (*p* < 0.05) among three different concentrations of the same sample, and lowercase letters (a,b) refer to significantly differences (*p* < 0.05) among samples at the same concentration.

Macrophages are key cells in innate immune responses that can kill pathogens directly by phagocytosis or indirectly by secreting bioactive molecules such as nitric oxide (NO), interleukin (IL)-6, IL-12, and tumor necrosis factor (TNF)-α [32]. Modulating these systems can significantly impact both humoral and cellular immune responses [19]. Here, the cytotoxic effects of PLE0s on RAW264.7 cells were examined at concentrations of 1–100 μg/mL (Figure 3A). Compared with the negative control (NC), the viabilities of cells treated with S1-PLE0, S2-PLE0, and S3-PLE0 were >100% at all doses tested, showing a similar trend for the three PLE0 samples and indicating that treatment with all PLE0s at doses of 10–100 µg/mL promoted macrophage proliferation. Thus, the results suggested that the PLE0s were not cytotoxic to RAW264.7 macrophages, but rather could stimulate their activation.

Compared to non-activated macrophages, several distinguishing features were observed in activated macrophages, such as phagocytosis and engulfment and destruction of foreign substances [33]. After phagocytosis, macrophages become antigen-presenting cells and interact with lymphocytes to regulate adaptive immune responses [4]. Therefore, the effects of three PLE0s on the phagocytic activity of RAW264.7 cells were evaluated using a neutral red phagocytosis assay. Neutral red internalization increased significantly after treatment with S1-PLE0, S2-PLE0, or S3-PLE0 (1–100 μg/mL) compared to the negative control (*p* < 0.05; Figure 3B). At 100 μg/mL, the phagocytic indices of S1-PLE0, S2-PLE0, and S3-PLE0 were 1.72, 2.41, and 1.95, respectively. S2-PLE0 exhibited much stronger phagocytic activity than S1-PLE0 and S3-PLE0. These results suggested that all three PLE0s could activate, trigger, and increase phagocytosis in macrophages. In particular, S2-PLE0 treatment was the most potent enhancer of macrophage activation.

#### 2.2.2. Effects on NO and Cytokine Production

**Figure 4:**
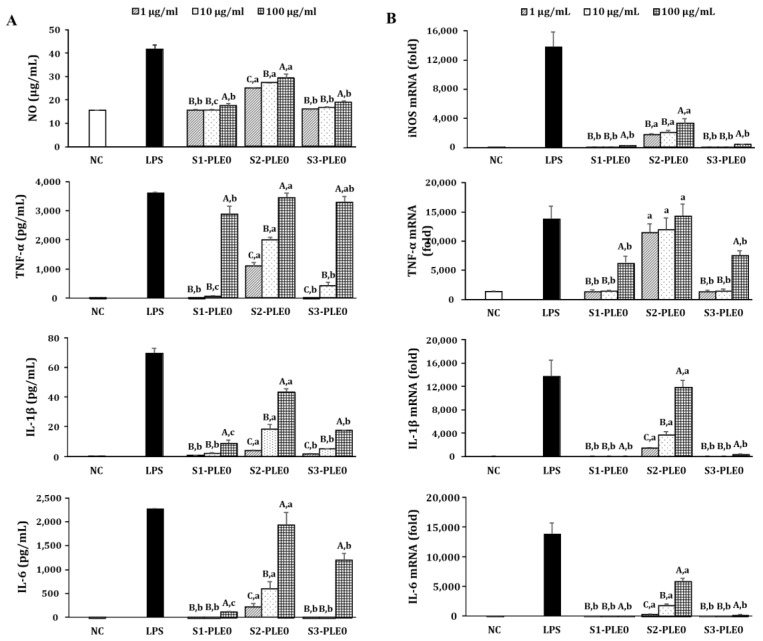
Effects of PLE0s extracted from persimmon leaf at three different maturity stages on nitric oxide (NO), TNF-α, IL-1β, and IL-6 production in RAW264.7 macrophages. RAW264.7 cells were treated with various concentrations of the polysaccharide fractions for 24 h. (**A**) NO levels in the culture media were determined by measuring nitrite accumulation, and the secretion levels of TNF-α, IL-1β, and IL-6 were measured by ELISA. (**B**) The mRNA-expression levels of macrophage-activating factors such as iNOS, TNF-α, IL-1β, and IL-6 were quantified by qRT-PCR. Medium alone was used as the NC, and LPS alone (1 µg/mL) was used as the positive control. Uppercase letters (A–C) indicate significant differences (*p* < 0.05) among samples at three different concentrations, and lowercase letters (a–c) indicate significant differences (*p* < 0.05) among samples at the same concentration.

It is well known that macrophage activation by immunomodulators can enhance the secretion of various bioactive molecules including NO, cytokines, and chemokines, which play pivotal roles in the immune system, such as the regulation of the balance between humoral and cell-based immune responses [5,6]. In this study, the effects of three different PLE0s on NO production by RAW264.7 cells were evaluated at concentrations of 1–100 μg/mL. S1-PLE0, S2-PLE0, and S3-PLE0 significantly enhanced NO secretion from the cells (*p* < 0.05; Figure 4A). Among them, S2-PLE0 showed significant stimulant activity (*p* < 0.05). In contrast, S1-PLE0 exhibited the lowest NO-releasing capacity. Additionally, we investigated by quantitative reverse transcription–polymerase chain reaction (qRT-PCR) analysis whether increased NO secretion was due to transcriptional enhancement of the iNOS gene (Figure 4B). S2-PLE0 induced iNOS mRNA expression, which may explain the increased NO secretion from macrophages after S2-PLE0 exposure. Similar results were observed regarding the secretion of cytokines such as TNF-α, IL-1β, and IL-6 from RAW264.7 cells and their gene expression levels. Compared with untreated cells, the three polysaccharides (S1-PLE0, S2-PLE0, and S3-PLE0) significantly stimulated cytokine secretion in a dose-dependent manner (Figure 4A). Of note, S2-PLE0 was the most effective over the concentrations tested and dramatically promoted cytokine secretion from RAW264.7 cells. Indeed, the cytokine levels after treatment with S2-PLE0 at 100 µg/mL were comparable to those after stimulation with the positive control, lipopolysaccharide (LPS.) Additionally, S2-PLE0 enhanced the mRNA-expression levels of TNF-α, IL-1β, and IL-6, consistent with the changes we observed in their secretion (Figure 4B). Particularly, S2-PLE0 induced considerably high TNF-α mRNA expression in RAW264.7 cells at the concentrations tested, with TNF-α expression after treatment with 100 µg/mL S2-PLE0 exceeding that of the LPS group (*p* < 0.05). Macrophage activation can be regulated by immune-related genes [7]. Collectively, these results suggest that PLE0 exhibited significant immunostimulating activities by promoting NO and cytokine secretion through the upregulation of their expression levels in activated macrophages.

**Figure 5:**
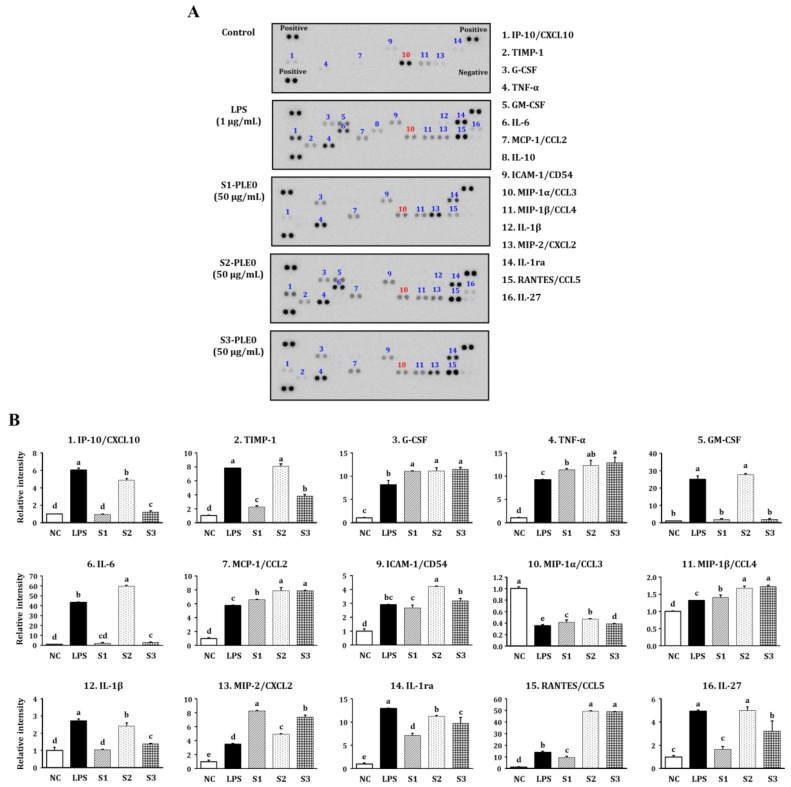
Effects of PLE0s extracted from persimmon leaf at three different maturity stages on the cytokine secretion profiles of RAW264.7 macrophages. (**A**) The levels of cytokines and chemokines in the culture media were determined with cytokine arrays after the cells were treated with vehicle control, 1 µg/mL LPS, or 50 μg/mL of S1-PLE0, S2-PLE0, or S3-PLE0. Blue numbers indicate increased cytokine and chemokine levels compared with the NC, whereas red numbers indicate decreased chemokine levels. (**B**) The relative level of each cytokine or chemokine was calculated using Image J software, version 1.8.0_66 (National Institutes of Health, Bethesda, MD, USA). The data shown are presented as the mean ± standard deviation (SD), and lowercases letters (a–e) indicate significant differences (*p* < 0.05) among five independent groups.

Further experiments on the effects of PLE0 on the cytokine-secretion profile of RAW264.7 cells were conducted using a cytokine antibody array for 16 different cytokines, including TNF-α, IL-1β, IL-6, IL-10, IL-27, interleukin-1 receptor antagonist (IL-1ra), interferon inducible protein-10 (IP-10), tissue inhibitor of metalloproteinases-1 (TIMP)-1, granulocyte colony-stimulating factor (G-CSF), granulocyte-macrophage colony-stimulating factor (GM-CSF), monocyte chemotactic protein (MCP)-1, intercellular adhesion molecule (ICAM)-1, macrophage inflammatory protein (MIP)-1α, MIP-1β, MIP-2, and regulated on activation, normal T cell expressed and secreted (RANTES). Treatment with LPS (the positive control) enhanced the secretion of 15 different cytokines compared to the control, except for MIP-1α (*p* < 0.05; Figure 5). S1-PLE0, S2-PLE0, and S3-PLE0 could also enhanced the secretion of various cytokines and chemokines, such as TNF-α, IL-27, IL-1ra, TIMP-1, G-CSF, MCP-1, ICAM-1, and RANTES. These cytokines and chemokines are produced by various types of cells and serve vital roles in immune cell development, activation, differentiation, regulation, functions, and homeostasis, in both innate and adaptive immunity [34]. However, their levels were significant different after treatment with the three PLE0 fractions, and their effects on cytokine production in macrophages were ranked as follows: S2-PLE0 > S3-PLE0 > S1-PLE0. Here, S2-PLE0 treatment led to marked elevation in the secretion of various cytokines, consistent with the results presented above. IL-10 secretion from RAW264.7 cells was not affected, and MIP-1α was downregulated by PLE0 treatment, suggesting that these polysaccharides also have immuno-modulating effects. Overall, S2-PLE0-treated cells showed a greater induction of cytokine production and exhibited a similar cytokine profile compared with cell treated with the positive control LPS. These data also suggest that seasonal variation in persimmon leaves can significantly affect the immunostimulatory activities of the polysaccharides.

## 3. Materials and Methods

### 3.1. Materials and Chemicals

Persimmon (*D. kaki* Thumb.) leaves were raised and collected from Dongsang-myeon (Wanju, Jeonbuk, Korea), which is the representative persimmon production region in Korea. The leaves were harvested in 2015 at three different maturity stages, after which they were washed and air-dried (Figure S2): (1) early June (young stage; designated as S1) when the plant begins to bear fruit and is considered suitable for commercial tea, (2) early August (early green mature stage; designated as S2), when the leaves have almost reached their final size as the fruit begins to grow, and (3) early October (full mature stage; designated as S3) when the fruit is completely ripe and suitable for immediate consumption [10].

A commercial pectinase from *Aspergillus niger* (Plantase MAX) was purchased from Vision Corporation (Seongnam, Gyonggi, Korea). The RAW264.7 murine macrophage cell line was obtained from the Korean Cell Line Bank (Seoul, Korea), and Dulbecco’s modified Eagle’s medium (DMEM), fetal bovine serum (FBS), and penicillin/streptomycin were purchased from Gibco BRL Co. (Grand Island, NY, USA). The pullulan standard set, LPS, and Griess reagent were purchased from Sigma (St. Louis, MO, USA). The enzyme-linked immunosorbent assay (ELISA) kits for TNF-α and IL-6 were obtained from BD Bioscience (San Diego, CA, USA), and an IL-1β ELISA kit was obtained from R&D Systems (Minneapolis, MN, USA). The Proteome Profiler™ Mouse Cytokine Array kit was purchased from R&D Systems (Minneapolis, MN, USA). All other chemicals were of analytical grade.

### 3.2. Preparation of Polysaccharides from Persimmon Leaves at Different Maturity Stages

Dried persimmon leaves (500 g) at different maturity stages were ground and dissolved in distilled water (10 L) and hydrolyzed using pectinase at 50 °C after adjustment to an initial pH of 4.5. After 48 h, pectinase was deactivated by boiling at 100 °C for 15 min. The pectinase hydrolysate of persimmon leaves was centrifuged at 6000 rpm for 20 min to remove insoluble precipitates. The supernatant was precipitated by adding three volumes of cold 99% ethanol to obtain a crude polysaccharide. The resulting precipitate was collected, dissolved in distilled water, and dialyzed using a Spectra/Por dialysis membrane (molecular weight cut-off of 6–8 kilodaltons; Spectrum Laboratories Inc., Rancho Dominguez, CA, USA). Finally, the dialyzed solution was lyophilized to obtain crude polysaccharide fractions (PLE0) from persimmon leaves: S1-PLE0, a polysaccharide fraction obtained from persimmon leaves at a young stage (PL-S1); S2-PLE0, a polysaccharide fraction obtained from persimmon leaves at an early green mature stage (PL-S2); and S3-PLE0, a polysaccharide fraction obtained from persimmon leaves at a fully mature stage (PL-S3).

### 3.3. Chemical Component Analysis of Polysaccharides

Total neutral sugar, uronic acid, KDO-like materials, and protein contents were determined by the phenol–sulfuric acid method [35], the *m*-hydroxybiphenyl method [36], the modified thiobarbituric acid (TBA)-positive method [37], and the Bradford method [38], respectively, using glucose, galacturonic acid, KDO, and bovine serum albumin as the respective standards. Monosaccharide compositions were analyzed using HPAEC coupled with pulsed amperometric detection (ICS-5000, Dionex Co., Sunnyvale, CA, USA). Briefly, the samples were hydrolyzed with 2 M trifluoroacetic acid (TFA) at 100 °C for 4 h. After TFA removal, the monosaccharides in the hydrolysate were separated on a CarboPac PA-1 analytical column (250 mm × 4 mm; Dionex Co., Sunnyvale, CA, USA) at 25 °C. Neutral sugars were eluted with 18 mM NaOH, and uronic acids were eluted with 100 mM NaOAc in 100 mM NaOH for 30 min at a constant flow rate of 1.0 mL/min. Arabinose, fucose, galactose, glucose, mannose, rhamnose, xylose, galacturonic acid, and glucuronic acid were used as monosaccharide standards.

### 3.4. Estimating the Molecular Weights of Polysaccharides

To determine the molecular weight patterns of the polysaccharide samples, HPSEC was performed using a JASCO PU-2089 Plus system equipped with Asahipak GS-520 and GS-320 columns (0.76 × 30 cm each; Showa Denko, Co. Ltd., Tokyo, Japan) and a refractive index detector (JASCO RI-2031 Plus, Jasco, Tokyo, Japan). A solution of PLE0 (15 mg/mL, 20 μL) was eluted with an isocratic buffer (50 mM ammonium formate buffer, pH 5.5) at a flow rate of 0.4 mL/min. The molecular weights were estimated from the calibration curve generated using standard pullulan samples (P-336, 113, 48.8, 21.7, 10, 6, 1.32, and 0.342 kDa; line equation: log molecular weight = −0.1263 RT + 8.4948, R^2^ = 0.9965).

### 3.5. FT-IR Analysis

The FT-IR spectra of the polysaccharides were determined using a Fourier-transform infrared spectrophotometer (FTIR-4600; Jasco, Tokyo, Japan). The polysaccharide samples were ground with KBr powder and then pressed into 1 mm pellets for FT-IR measurement in the frequency range of 4000–500 cm^−1^ to detect functional groups.

### 3.6. Immunostimulatory Activities of Polysaccharides

#### 3.6.1. Cell Culture and Cytotoxicity Assays

RAW264.7 macrophages were cultured in DMEM containing 10% heat-inactivated FBS, penicillin (100 U/mL), and streptomycin (100 μg/mL). The cells were incubated at 37 °C under 5% CO_2_. The effects of PLE0 on RAW264.7 cell viability were evaluated using a conventional Cell Counting Kit-8 (CCK-8; Cell Counting Kit, Dojindo, Tokyo, Japan) assay. Briefly, RAW264.7 cells (2 × 10^5^ cells/mL) were plated in 96-well plates for 24 h and then treated with different concentrations of PLE0 (1–100 μg/mL) or LPS (1 μg/mL) for additional 24 h. Subsequently, 10 µL/well of CCK-8 solution was added to the cultured cells, and the cytotoxic effects were determined, per the manufacturer’s instructions. The viability of treated cells was expressed as a percentage of that of negative control cells treated with medium alone.

#### 3.6.2. Phagocytic Activity

The effects of PLE0s on the phagocytic activity of macrophages were measured by performing neutral red assays. Macrophages (2 × 10^5^ cells/mL) were treated with the PLE0 samples (1–100 μg/mL) or LPS, as mentioned above. Then, the medium was removed, 100 μL of 0.1% neutral red was added to each well, and the cells were incubated for 30 min. After washing three times with PBS, 100 μL of cell-lysing solution (1 M glacial acetic acid/ethanol = 1:1) was added to each well, and the plates ere incubated for 1 h at room temperature without movement. The absorbance was measured at 540 nm using a microplate reader, and the phagocytic index was calculated using the following equation: Abs _sample_/Abs _blank control_.

#### 3.6.3. Measurement of NO, TNF-α, IL-1β, and IL-6 Cytokine Secretion

After treatment with the PLE0 samples (1–100 μg/mL) or LPS (1 μg/mL) for 24 h as described above, each culture supernatant was collected to quantify NO, TNF-α, IL-1β, and IL-6 secretion from the macrophages. The NO content in the culture supernatant was measured on the basis of the Griess method, and the levels of TNF-α, IL-1β, and IL-6 were determined using a corresponding ELISA set (BD Biosciences, Pharmingen, San Diego, CA, USA), according to the manufacturer’s instructions.

#### 3.6.4. qRT-PCR Analysis

Total RNA was isolated from RAW264.7 cells treated with the PLE0 samples (1–100 μg/mL) using NucleoSpin RNA (Macherey-Nagel, Duren, Germany) and then reversed into cDNA using ReverTra Ace qPCR RT Master Mix (Toyobo, Osaka, Japan), according to the manufacturer’s protocol. To amplify the cDNA, the reverse-transcribed cDNA was subjected to 40 cycles of qRT-PCR using the SYBR Green Real-Time PCR Master Mix (Toyobo, Osaka, Japan) in a CFX96 Touch Real-Time PCR instrument (Bio-Rad, Hercules, CA, USA). The nucleotide sequences of the specific primers are shown in Table 2. GAPDH mRNA was detected as the internal reference. Fold-changes in the mRNA expression levels of iNOS, TNF-α, IL-1β, and IL-6 were determined relative to the control GAPDH gene expression using the 2^−ΔΔ^Ct method.

#### 3.6.5. Cytokine Array

PLE0-induced changes in the cytokine-secretion profile from RAW264.7 macrophages were assessed using a cytokine antibody array. The cells were stimulated with the PLE0 samples (50 μg/mL) or LPS (1 μg/mL) for 24 h, and the supernatants were collected. Cytokine production was analyzed using a Proteome Profiler™ Mouse Cytokine Array Panel A Kit (R&D Systems, Minneapolis, MN, USA), per the manufacturer’s protocol. Briefly, cytokine array membranes were incubated with a blocking buffer for 1 h, followed by overnight incubation with the culture supernatants and an antibody cocktail. The membranes were then washed and incubated with Streptavidin-HRP for 30 min. Finally, the chemiluminescent signals were detected using a ChemiDoc XRS system (Bio-Rad, Hercules, CA, USA).

### 3.7. Statistical Analysis

All experiments were performed in triplicate. The data were expressed as the mean ± the standard deviation. Statistically significant differences among groups were evaluated via factorial analysis of variance (one-way analysis of variance) with Duncan’s post-hoc test; *p*-values < 0.05 were regarded as reflecting significant differences. All statistical analyses were performed using the SPSS statistical package, version 20 (SPSS Inc., Chicago, IL, USA).

## 4. Conclusions

The purpose of this study was to determine changes in the structural and biological features of polysaccharides extracted from persimmon leaves at different growth stages. Here, we confirmed that their physicochemical and immunostimulatory properties varied significantly during different maturity stages. The most noticeable differences occurred in the immunostimulatory effects, with respect to which S2-PLE0 was clearly the most effective, followed by S3-PLE0 and then S1-PLE0. These differences in the three polysaccharides might be attributed to their different physicochemical characteristics, including differences in their sugar compositions, molecular weight distributions, and types of linkages. Thus, the high immunostimulatory activity of S2-PLE0 might be related to its higher content of galactose, arabinose, rhamnose, and xylose; the presence of β-glucopyranoside linkages; and probably a more abundant or complex branched region in the pectic structure. The polysaccharide yields were lower during the later growth stages of permission leaves, which might reflect a reduction of their solubility after polymerization, but this possibility needs to be confirmed. Overall, the results of this study suggest that persimmon leaves from plants in the early green mature stage are most desirable for obtaining immunostimulatory polysaccharides. In contrast, the younger stage, which is considered suitable for making commercial tea because of the leaves’ high phytochemical content (including polyphenols), might not be suitable for extracting immunostimulatory polysaccharides from persimmon leaves. The findings generated in this study strongly indicate that the selection of the appropriate maturity stage should be considered when exploring ways to obtain polysaccharides with enhanced functionality.

## Figures and Tables

**Table 1:** Chemical and monosaccharide compositions of polysaccharide fractions (PLE0s) extracted from persimmon leaves at three different maturity stages.

Samples	S1-PLE0	S2-PLE0	S3-PLE0
Yield (%) ^1^	1.80 ± 0.23 ^a^	1.44 ± 0.18 ^ab^	1.20 ± 0.24 ^b^
Chemical composition (%)			
Neutral sugar	65.3 ± 3.6 ^a^	57.0 ± 2.7 ^b^	62.5 ± 3.9 ^ab^
Uronic acid	32.9 ± 3.4 ^b^	40.9 ± 3.4 ^a^	35.2 ± 2.6 ^ab^
Protein	0.6 ± 0.1 ^b^	0.8 ± 0.0 ^ab^	0.9 ± 0.1 ^a^
KDO ^2^-like material	1.2 ± 0.1	1.4 ± 0.3	1.3 ± 0.1
Sugar composition (molar ratio)			
Galactose	2.06	4.46	4.07
Arabinose	0.60	1.59	0.94
Glucose	1.00	1.00	1.00
Rhamnose	0.31	0.75	0.11
Mannose	0.20	0.35	0.41
Xylose	0.16	0.30	0.09
Fucose	0.03	0.09	0.04
Galacturonic acid	7.78	10.94	3.95
Glucuronic acid	0.24	0.43	0.45

Values are means ± SD, *n* = 3. Values with different small letters (a,b) in the same column are significantly different (*p* < 0.05). ^1^ Yield (%) based on dry materials. ^2^ KDO, 2-keto-3-deoxy-d-manno-octulosonic acid.

**Table 2:** Primer sequences used for real-time quantitative reverse transcription PCR (qRT-PCR).

Gene	Forward Primer	Reverse Primer
iNOS	5′-CCAGCCTGCCCCTTCAAT-3′	5′-ATCCTTCGGCCCACCTTCCT-3′
TNF-α	5′-AGGCACTCCCCCAAAAGATG-3′	5′-CACCCCGAAGTTCAGTAGACAGA-3′
IL-1β	5′-TGACGGACCCCAAAAGAT-3′	5′-GTGATACTGCCTGCCTGAAG-3′
IL-6	5′-CCGGAGAGGAGACTTCACAGAG-3′	5′-TCATTTCCACGATTTCCCAGAG-3′
GAPDH	5′-CATGGCCTTCCGTGTTCCTAC-3′	5′-TCAGTGGGCCCTCAGATGC-3′

## Supplementary Materials

The following are available online, Figure S1: The HPAEC-PAD profile on monosaccharide component analysis of PLE0s extracted from persimmon leaves at three different maturity stages, Figure S2: Persimmon leaves at three different maturity stages.

## References

[1] Yu Y., Shen M., Song Q., Xie J. (2018). Biological activities and pharmaceutical applications of polysaccharide from natural resources: A review. Carbohydr. Polym..

[2] Liu J., Willför S., Xu C. (2015). A review of bioactive plant polysaccharides: Biological activities, functionalization, and biomedical applications. Bioact. Carbohydr. Dietary Fibre.

[3] Chen Y., Jiang X., Xie H., Li X., Shi L. (2018). Structural characterization and antitumor activity of a polysaccharide from *Ramulus mori*. Carbohydr. Polym..

[4] Nie C., Zhu P., Ma S., Wang M., Hu Y. (2018). Purification, characterization and immunomodulatory activity of polysaccharides from stem lettuce. Carbohydr. Polym..

[5] Wang M., Yang X.B., Zhao J.W., Lu C.J., Zhu W. (2017). Structural characterization and macrophage immunomodulatory activity of a novel polysaccharide from *Smilax glabra* Roxb. Carbohydr. Polym..

[6] Ferreira S.S., Passos C.P., Madureira P., Vilanova M., Coimbra M.A. (2015). Structure–function relationships of immunostimulatory polysaccharides: A review. Carbohydr. Polym..

[7] Wu F., Zhou C., Zhou D., Ou S., Huang H. (2017). Structural characterization of a novel polysaccharide fraction from *Hericium erinaceus* and its signaling pathways involved in macrophage immunomodulatory activity. J. Funct. Foods.

[8] Vannucci L., Krizan J., Sima P., Stakheev D., Caja F., Rajsiglova L., Horak V., Saieh M. (2013). Immunostimulatory properties and antitumor activities of glucans. Int. J. Oncol..

[9] Pérez-Burillo S., Oliveras M.J., Quesada J., Rufián-Henares J.A., Pastoriza S. (2017). Relationship between composition and bioactivity of persimmon and kiwifruit. Food Res. Int..

[10] Xie C., Xie Z., Xu X., Yang D. (2015). Persimmon (*Diospyros kaki* L.) leaves: A review on traditional uses, phytochemistry and pharmacological properties. J. Ethnopharmacol..

[11] Sakanaka S., Tachibana Y., Okada Y. (2005). Preparation and antioxidant properties of extracts of Japanese persimmon leaf tea (kakinoha-cha). Food Chem..

[12] Kawakami K., Aketa S., Nakanami M., Iizuka S., Hirayama M. (2010). Major water-soluble polyphenols, proanthocyanidins, in leaves of persimmon (*Diospyros kaki*) and their α-amylase inhibitory activity. Biosci. Biotechnol. Biochem..

[13] Raza A., Li F., Xu X., Tang J. (2017). Optimization of ultrasonic-assisted extraction of antioxidant polysaccharides from the stem of *Trapa quadrispinosa* using response surface methodology. Int. J. Biol. Macromol..

[14] Chen L., Wei Y., Zhao S., Zhang M., Yan X., Gao X., Li J., Gao Y., Zhang A., Gao Y. (2018). Antitumor and immunomodulatory activities of total flavonoids extract from persimmon leaves in H 22 liver tumor-bearing mice. Sci. Rep..

[15] Thuong P.T., Lee C.H., Dao T.T., Nguyen P.H., Kim W.G., Lee S.J., Oh W.K. (2008). Triterpenoids from the leaves of Diospyros kaki (persimmon) and their inhibitory effects on protein tyrosine phosphatase 1B. J. Nat. Prod..

[16] Kawakami K., Shibukura Y., Kanno T., Furuki T., Aketa S., Hirayama M. (2011). Identification of 2″-galloylated flavonol 3-O-glycosides accumulating in developing leaves of persimmon. Phytochem. Anal..

[17] Duan J., Dong Q., Ding K., Fang J. (2010). Characterization of a pectic polysaccharide from the leaves of Diospyros kaki and its modulating activity on lymphocyte proliferation. Biopolymers.

[18] Deng H., He M., Li J., Luo X.Y., Huang R. (2011). Hypoglycemic effect of persimmon leaf polysaccharide in diabetic mice induced by streptozotocin. Chin. J. Exp. Tradit. Med. Formulae.

[19] Kim H., Hong H.D., Suh H.J., Shin K.S. (2016). Structural and immunological feature of rhamnogalacturonan I-rich polysaccharide from Korean persimmon vinegar. Int. J. Biol. Macromol..

[20] Park H.R., Hwang D., Hong H.D., Shin K.S. (2017). Antitumor and antimetastatic activities of pectic polysaccharides isolated from persimmon leaves mediated by enhanced natural killer cell activity. J. Funct. Foods.

[21] Lee S.G., Jung J.Y., Shin J.S., Shin K.S., Cho C.W., Rhee Y.K., Hong H.D., Lee K.T. (2015). Immunostimulatory polysaccharide isolated from the leaves of *Diospyros kaki* Thumb modulate macrophage via TLR2. Int. J. Biol. Macromol..

[22] Shin M.S., Lee H., Hong H.D., Shin K.S. (2016). Characterization of immunostimulatory pectic polysaccharide isolated from leaves of *Diospyros kaki* Thumb. (Persimmon). J. Funct. Foods.

[23] Zhu C., Liu X. (2013). Optimization of extraction process of crude polysaccharides from pomegranate peel by response surface methodology. Carbohydr. Polym..

[24] Liu Y., Fang S., Zhou M., Shang X., Yang W., Fu X. (2018). Geographic variation in water-soluble polysaccharide content and antioxidant activities of *Cyclocarya paliurus* leaves. Ind. Crops Prod..

[25] Ghasemzadeh A., Jaafar H.Z., Ashkani S., Rahmat A., Juraimi A.S., Puteh A., Muda Mohamed M.T. (2016). Variation in secondary metabolite production as well as antioxidant and antibacterial activities of *Zingiber zerumbet* (L.) at different stages of growth. BMC Complement. Altern. Med..

[26] Jeon D.B., Hong Y.S., Lee G.H., Park Y.M., Lee C.M., Nho E.Y., Choi J.Y., Jamila N., Khan N., Kim K.S. (2017). Determination of volatile organic compounds, catechins, caffeine and theanine in Jukro tea at three growth stages by chromatographic and spectrometric methods. Food Chem..

[27] Jung W.Y., Jeong J.M. (2012). Change of antioxidative activity at different harvest time and improvement of atopic dermatitis effects for persimmon leaf extract. Korea J. Herbol..

[28] Patova O.A., Golovchenko V.V., Ovodov Y.S. (2014). Pectic polysaccharides: Structure and properties. Russ. Chem. Bull..

[29] Kacurakova M., Capek P., Sasinkova V., Wellner N., Ebringerova A. (2000). FT-IR study of plant cell wall model compounds: Pectic polysaccharides and hemicelluloses. Carbohydr. Polym..

[30] Wang X.Y., Yin J.Y., Nie S.P., Xie M.Y. (2018). Isolation, purification and physicochemical properties of polysaccharide from fruiting body of *Hericium erinaceus* and its effect on colonic health of mice. Int. J. Biol. Macromol..

[31] Ognyanov M., Georgiev Y., Petkova N., Ivanov I., Vasileva I., Kratchanova M. (2018). Isolation and characterization of pectic polysaccharide fraction from in vitro suspension culture of *Fumaria officinalis* L.. Int. J. Polym. Sci..

[32] Wang M., Zhao S., Zhu P., Nie C., Ma S., Wang N., Du X., Zhou Y. (2018). Purification, characterization and immunomodulatory activity of water extractable polysaccharides from the swollen culms of *Zizania latifolia*. Int. J. Biol. Macromol..

[33] Sun Y., Gong G., Guo Y., Wang Z., Song S., Zhu B., Zhao L., Jiang J. (2018). Purification, structural features and immunostimulatory activity of novel polysaccharides from *Caulerpa lentillifera*. Int. J. Biol. Macromol..

[34] Kwak J.Y., Mamura M., Barlic-Dicen J., Grage-Griebenow E. (2014). Pathophysiological roles of cytokine-chemokine immune network. J. Immunol. Res..

[35] Dubois M., Gilles K.A., Hamilton J.K., Rebers P.T., Smith F. (1956). Colorimetric method for determination of sugars and related substances. Anal. Chem..

[36] Blumenkrantz N., Asboe-Hansen G. (1973). New method for quantitative determination of uronic acids. Anal. Biochem..

[37] Karkhanis Y.D., Zeltner J.Y., Jackson J.J., Carlo D.J. (1978). A new and improved microassay to determine 2-keto-3-deoxyoctonate in lipopolysaccharide of gram-negative bacteria. Anal. Biochem..

[38] Bradford M.M. (1976). A rapid and sensitive method for the quantitation of microgram quantities of protein utilizing the principle of protein-dye binding. Anal. Biochem..

