# Supplementary material for: Discrimination of Structural and Immunological Features of Polysaccharides from Persimmon Leaves at Different Maturity Stages
Source: Molecules. 2019 Jan 19;24(2):356. doi: 10.3390/molecules24020356 (PMC6359638; doi:10.3390/molecules24020356)
Supplement: Supplementary file 1 [file molecules-24-00356-s001.pdf]

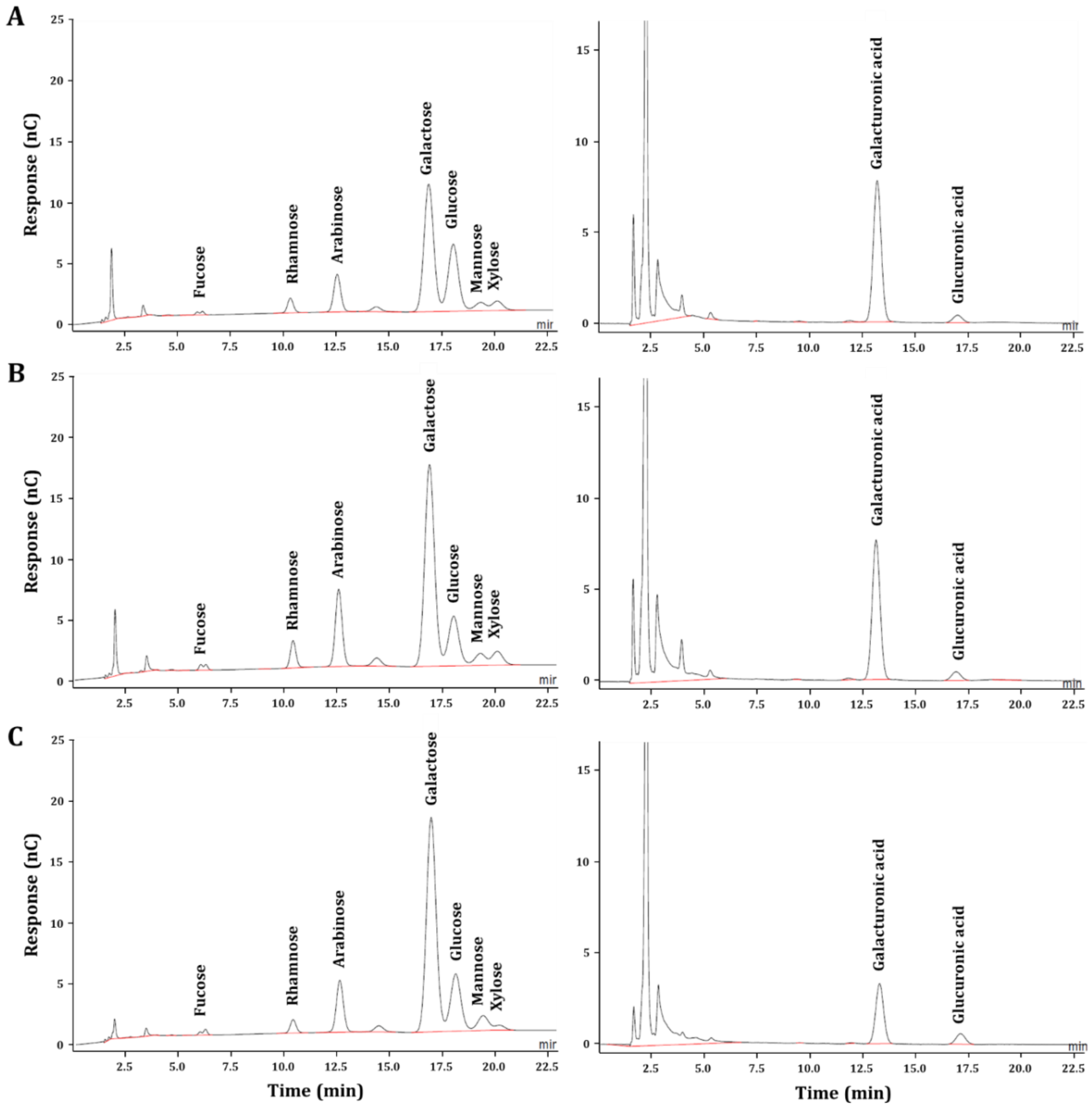


**Figure S1.** The HPAEC-PAD profile on monosaccharide component analysis of PLE0s extracted from persimmon leaves at three different maturity stages. (A) S1-PLE0, (B) S2-PLE0, and (C) S3-PLE0. Left side; the chromatograms on seven neutral sugars of fucose (6.00 min), rhamnose, (10.40 min), arabinose (12.62 min), galactose (16.95 min), glucose (18.12 min), mannose (19.42 min), and xylose (20.17 min). Right side; the chromatograms on two uronic acids of galacturonic acid (13.18 min), and glucuronic acid (17.00 min).

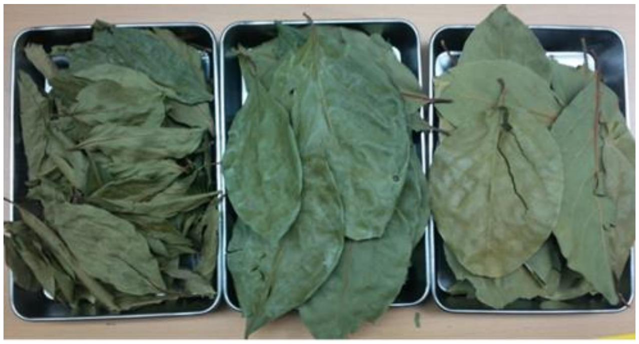

**PL-S1** **PL-S2** **PL-S3**

**Figure S2.** Persimmon leaves at three different maturity stages.
